# The Voice of Anger: Oscillatory EEG Responses to Emotional Prosody

**DOI:** 10.1371/journal.pone.0159429

**Published:** 2016-07-21

**Authors:** Renata del Giudice, Christine Blume, Malgorzata Wislowska, Tomasz Wielek, Dominik P. J. Heib, Manuel Schabus

**Affiliations:** 1 University of Salzburg, Department of Psychology, Laboratory for Sleep, Cognition and Consciousness Research, Hellbrunner Strasse 34, 5020, Salzburg, Austria; 2 Centre for Cognitive Neuroscience Salzburg (CCNS), University of Salzburg, Hellbrunner Strasse 34, 5020, Salzburg, Austria; Northwestern University, UNITED STATES

## Abstract

Emotionally relevant stimuli and in particular anger are, due to their evolutionary relevance, often processed automatically and able to modulate attention independent of conscious access. Here, we tested whether attention allocation is enhanced when auditory stimuli are uttered by an angry voice. We recorded EEG and presented healthy individuals with a passive condition where unfamiliar names as well as the subject’s own name were spoken both with an angry and neutral prosody. The active condition instead, required participants to actively count one of the presented (angry) names. Results revealed that in the passive condition the angry prosody only elicited slightly stronger delta synchronization as compared to a neutral voice. In the active condition the attended (angry) target was related to enhanced delta/theta synchronization as well as alpha desynchronization suggesting enhanced allocation of attention and utilization of working memory resources. Altogether, the current results are in line with previous findings and highlight that attention orientation can be systematically related to specific oscillatory brain responses. Potential applications include assessment of non-communicative clinical groups such as post-comatose patients.

## Introduction

Processing of emotional content of sensory stimuli has been demonstrated to be at least partially independent from voluntary top-down attention and has been shown to support bottom-up allocation of attentional resources. Several studies, also involving brain-damaged patients [1–3], have shown that humans are not able to fully ignore the emotional content of certain stimuli even if emotional processing is not explicitly required for the experiment (for a complete review see [4]). Therefore, emotional processing seems to be, at least to some extent, automatic and independent from conscious perception.

Beyond this, emotional stimulus characteristics have also been shown to modulate attention. Recent evidence clearly indicates that the emotional relevance of a stimulus can influence attention deployment and thus selection and prioritization of stimuli for further processing [5]. This has been suggested to result from their evolutionary and adaptive significance, due to which emotional stimuli are represented in a broader brain network and have preferential access to awareness [4, 6, 7]. More precisely, this facilitates perception, boosts attention and facilitates attention allocation as well as processing of subsequently occurring stimuli [4, 5, 8, 9]. From this perspective, anger seems to be particularly important because, despite its negative significance, it is an “approach-related” emotion. That is, it promotes the tendency to act and initiates goal-directed behaviour. In fact, as explained by Carver and Harmon-Jones [10] it is related to the approach-oriented motivational system functioning, and it is therefore, associated with higher levels of attention, facilitation of stimulus perception as well as prioritization of orienting towards significant stimuli [11, 12]. We suggest that these characteristics render emotional stimuli especially suitable for studies involving populations with difficulties to engage and maintain attention. Therefore, such stimuli could be particularly useful when assessing clinical groups such as patients suffering from disorders of consciousness (DOC). In this population the level of awareness and thus consciousness varies with the clinical diagnosis, and attentive processing is highly variable due to recurring arousal fluctuations [13, 14]. This variability renders clinical assessment challenging, which may lead to a high rate of misdiagnoses [15, 16] and greater difficulty in interpretation of negative findings regarding the level of awareness in DOC patients, i.e. we cannot conclude the absence of awareness from the absence of signs of awareness. In a former study from our group [17], we tested whether introducing self-relevant stimuli (i.e. the voice of a family member) in the classical own name paradigm might be able to facilitate attention allocation and therefore increase the chance of detecting signs of consciousness in DOC patients using an active-passive paradigm [18–22]. However, practically, the use of stimuli recorded with the voice of a family member is not always feasible, and it is difficult to record such material in a standardized manner (e.g., matching in prosody, stimulus duration, volume, etc.) in a clinical setting. Therefore, we intended to test a more economical version in the present study by introducing standardized stimulus material varying emotional prosody rather than familiarity of voice. To this end, we tested the so-called eSON (emotional subject’s own name) paradigm in healthy individuals with the aim to investigate whether the allocation of attention can be enhanced using angry voice stimuli. We presented healthy individuals with their own name and unfamiliar names spoken by and angry or a neutral prosody and asked them to passively listen (passive condition) or count (active condition) one of the presented names. We furthermore focus on the temporal dynamics of various EEG frequency bands and in particular delta, theta and alpha frequencies. The utilized time-frequency analysis benefits of less temporal dispersion as it accounts for evoked as well as induced activity as compared to classical event-related potential studies [23]; this is of specific advantage when planning to use this paradigm in DOC patients in the future [22, 24–26]. Furthermore, delta, theta and alpha oscillations have been consistently linked to specific functions such as attention, working memory and long-term memory [27–30] and therefore might allow a deeper insight into brain processing in healthy as well as clinical groups.

## Materials and Methods

### Subjects

EEG data were recorded from 31 healthy controls. We excluded data from five participants from subsequent data analyses due to technical reasons or because they scored abnormally high on self-evaluation scales for psychiatric symptoms. The remaining sample comprised 14 females and 12 males aged 19 to 54 (M = 25.69; SD = 6.65). All volunteers were recruited at the University of Salzburg with flyers or via cloud-based participant management software. All participants were right-handed native German speakers without any prior history of neurological or psychiatric disease. Participants gave written informed consent approved by the local ethics committee (University of Salzburg; EK-GZ 16/2014) and received monetary compensation or course credit for their participation. The data from the passive condition of a part of the sample included in the current study (14 out of 26 subjects) have previously been analysed in another study focusing on the effects the presentation of emotional stimulus material during sleep (cf. [31]).

### Experimental Design and Procedure

The aim of this study was to test a modified version of the classical “subject’s own name paradigm”, which we have been using in previous work from our group (for details please see [17]) introducing an angry voice instead of the voice of a family member. The subject’s own name (SON) and five commonly used Austrian names matched for the number of syllables (according to STATISTIK AUSTRIA; http://www.statistik.at/), length and gender were used. Stimuli were presented via headphones at a sound pressure level of approximately 65db. The SONs as well as the unfamiliar names (UNs) were recorded by a professional native German speaker and were either spoken with an angry or neutral prosody (angry voice, AV and neutral voice, NV). The professional speaker was simply instructed to speak naturally with more or less the same loudness as if addressing someone with an angry intonation. The names were spoken in six different prosody intensities with the first one being the neutral intonation and angry intonation increasing linearly from recordings two to six. Usually, the first and the sixth stimulus were selected as neutral and angry voice stimuli, respectively. Stimuli were furthermore manually inspected and pre-processed (denoising, normalisation) using Audacity software (http://audacityteam.org/) in order to render the stimuli comparable in length, loudness and background noise. For an example of the auditory stimuli please see S4 Fig. The task consisted of a passive listening condition and a subsequent active counting condition. The two tasks took about twenty minutes with the passive condition lasting twelve minutes and the active condition six minutes. During the passive condition, participants were asked to just listen to the names presented, while the active condition required them to focus on and silently count the number of occurrences of a specific target name. In the passive condition, the SON plus two different UNs, all uttered by an angry and a neutral voice (i.e. six stimuli in total), were presented. In the active condition, only unfamiliar names were presented and all of them were uttered by an angry voice giving rise to a total number of three different stimuli. In both conditions, each stimulus was presented 39 times. The inter stimulus interval [ISI] was set to 2000ms and Presentation® (Version 0.71; Presentation Software, Neurobehavioral Systems Inc., California) was used for stimulus presentation and synchronization.

### Data Acquisition

The EEG was recorded using 22 goldcup scalp electrodes with BrainAmp EEG amplifier (BrainProducts® GmbH, Gilching, Germany) and the Brain Vision Recorder software (Brain Products®). The sampling rate was set to 500 Hz and impedances were kept below 5kΩ. The setup consisted of 14 scalp, four electrooculogram (EOG) (two vertical and two horizontal) and two electromyogram (EMG) electrodes. Scalp positions were F3, Fz, F4, FC3, FC4, C3, Cz, C4, CP5, CPz, CP6, P3, Pz and P4, placed according to the international 10–20 system. Electrodes for subsequent re-referencing were placed on left and right mastoids.

### Data Analysis

Data pre-processing was done using Brain Vision Analyzer 2.0 (Brain Products, Gilching, Germany). In a first step we re-referenced data to digitally linked mastoids and bandpass filtered the data between 0.1 and 70 Hz with an additional notch filter set to 50Hz. Ocular correction was conducted using the regression-based approach from Gratton, Coles [32] as implemented in Brain Vision Analyzer 2.0 (Brain Products, Gilching, Germany). Afterwards, data were visually checked for further artefacts and segmented into long (overlapping) epochs ranging from -5000 to +4000 ms relative to stimulus onset. The length of the segments was selected to have a better time resolution and to allow a reliable estimation of the low frequencies. This procedure resulted in an average of 33 artefact-free trials for the active (T = 33, ± 3.5; NT = 32, ± 3.6) and 35 for the passive condition (AV = 35, ± 2.9; NV = 35, ± 3.6). EEG segments for each condition of interest were then exported and further analysed using lab-internal Matlab routines (MathWorks, Natick, MA) and the EEGLAB toolbox [33].

To characterize oscillatory activity, we used the event-related spectral perturbation (ERSP) method as implemented in EEGLAB. The ERSP measures the time course of a stimulus-induced change in spectral power of a predefined frequency range from pre-occurrence to post-occurrence of an experimental stimulus [33], and it is comparable with the classical ERS/ERD method [34]. Note that positive values reflect an increase in oscillatory activity from before to after stimulus onset, whereas negative values reflect a decrease. Event-related changes in spectral EEG power from before to after stimulus appearance were calculated for frequencies ranging from 1 to 30 Hz. ERSP computation was performed using two-cycle Morlet wavelets procedure with an increasing factor of 0.8. As a reference period, we chose the time bin from -700 to -200 ms before stimulus onset. Baseline correction was computed using the subtraction method by Hu, Xiao [35]. This approach is unbiased and allows minimizing the dominance of low frequency EEG power.

Total power was calculated in decibels (dB) relative to the baseline in each frequency band.

To increase comparability across studies, we focused on the same frequency bands previously analysed in del Giudice et al.,r [17]: delta (1–4 Hz), theta (4–7 Hz) and alpha band (8-12Hz). Resulting ERSP values within these frequency ranges were averaged for four consecutive post-stimulus time windows (t1 = 0–200, t2 = 200–400, t3 = 400–600 and t4 = 600–800 ms relative to stimulus onset).

### Statistical Analyses

Statistical analyses were performed using Matlab’s Statistics toolbox and EEGLAB’s statistics toolkit. Statistical differences in ERSP values between different conditions were calculated by performing a Monte Carlo cluster-based permutation test (4000 randomizations). This approach is implemented in the Fieldtrip toolbox [36] and it is a widely-used technique to address the multiple comparisons problem for electrophysiological data [37]. The critical *p*-value for each cluster is calculated using the Monte Carlo estimate and the cluster is considered to be significantly different from the others when the empirically observed value exceeds the significance criterion (cluster-level alpha, here *p* < 0.05).

The cluster-based permutation test was computed on the following conditions of interest: factors NAME (SON vs UN) and VOICE (AV vs NV) in the passive condition and CONDITION (Target vs. Non-target) in the active condition.

## Results

### Delta ERS in the Passive Condition

Statistical analyses revealed a significant difference between angry and neutral voice in the factor VOICE from 200 to 400 ms at electrodes F3, Fz, F4, FC4 and C4 (Monte Carlo *p* = 0.0132) with the angry voice giving rise to stronger delta synchronisation as compared to the neutral voice. Although present on a descriptive level, (cf. S1 Fig, upper panel) no significant differences were evident for the factor NAME and the interactions with the factor NAME (cf. S2 and S3 Figs) using cluster-based statistics.

### Theta ERS in the Passive Condition

**Fig 1:**
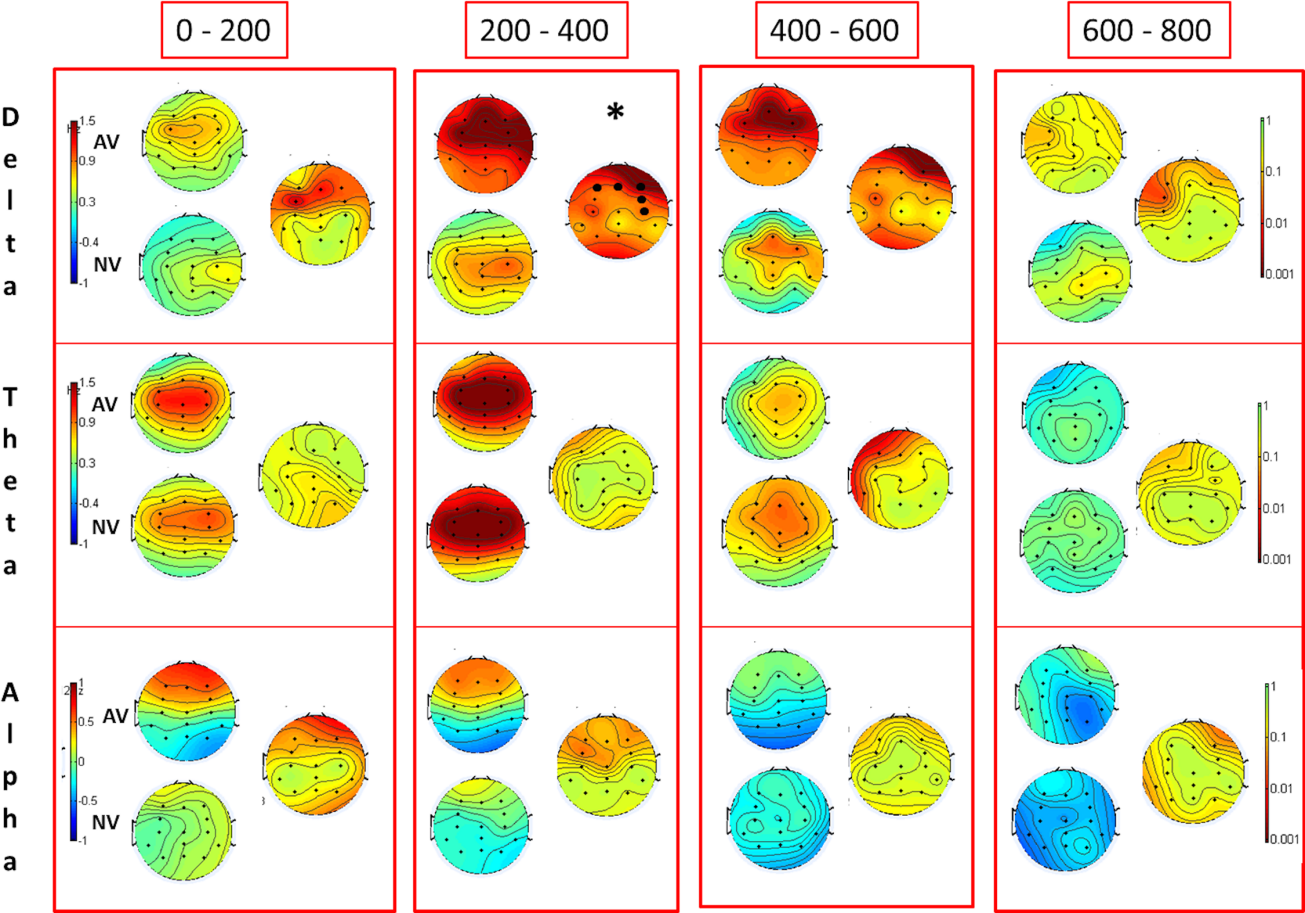
Topographic distribution of different frequencies during the passive condition. Topographic maps depict the topographic distribution for delta, theta and alpha frequency band during the passive condition from 0 to 800 ms after stimulus onset. Angry Voice (AV), Neutral Voice (NV) scalp distributions for the two conditions and p value distributions (right) are depicted. Note that bold black dots indicate electrodes significant after cluster correction and asterisks time windows where the effect was significant. Negative values indicate desynchronization (ERD) and positive ones synchronization (ERS) compared to baseline (from -700 to -200 ms before stimulus onset).

Also in the theta range no significant differences were evident for the factors VOICE (cf. Fig 1) and NAME (cf. S1 Fig), neither were significant interactions evident (cf. S2 and S3 Figs, middle panels) when applying cluster-based statistics.

### Alpha ERD in the Passive Condition

No statistical differences were observed for the factors VOICE and NAME and no significant interactions were found (cf. Fig 1 and S1–S3 Figs, lower panels).

### Delta ERS in the Active Condition

**Fig 2:**
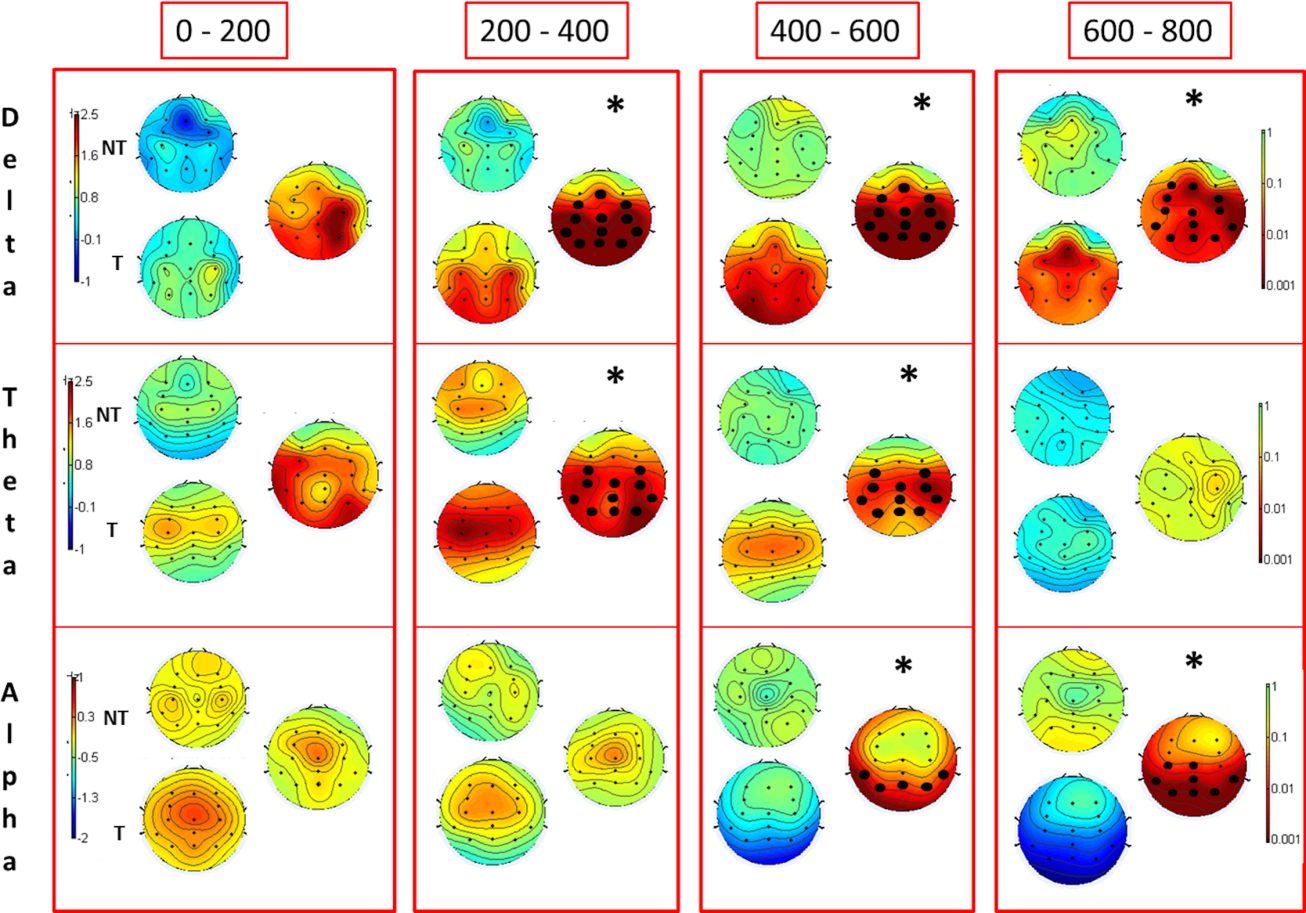
Topographic distribution of different frequencies during the active condition. Topographic maps depict the topographic distributions for delta, theta and alpha frequency band during the active condition from 0 to 800 ms after stimulus onset. Target (T), Non-Target (NT) scalp distributions as well as *p* value distributions (right) are depicted. Note that bold black dots indicate electrodes significant after cluster correction and asterisks time windows where the effect was significant. Negative values indicate desynchronization (ERD) and positive values synchronization (ERS) compared to baseline (from -700 to -200 ms before stimulus onset).

Analyses indicated that the target (i.e. always an UN in angry prosody) induced stronger delta synchronization as compared to non-targets at Fz, FC3, FC4, C3, Cz, C4, CP5, CPz, CP6, P3, Pz and P4 from 200 to 400 ms (all Monte Carlo *p* < 0.001), from 400 to 600 ms (all Monte Carlo *p* < 0.001), and from 600 to 800 ms at electrodes F3, Fz, FC3, FC4, C3, Cz, C4, CP5, CPz, CP6, P3, Pz and P4 (Monte Carlo *p* = 0.027) (see Fig 2, top panel).

### Theta ERS in the Active Condition

The comparison between target and non-target in the theta range yielded significant differences in the time windows from 200 to 400 at FC3, FC4, C3, Cz, C4, CP5, CPz, CP6, P3, Pz and P4 (Monte Carlo *p* < 0.001) and from 400 to 600 ms at FC3, FC4, C3, Cz, C4, CP5, CPz, CP6, P3, Pz and P4 (Monte Carlo *p* = 0.006) indicating stronger theta synchronization after target presentation between 200 and 600 ms (cf. Fig 2, middle panel).

### Alpha ERD in the Active Condition

In the alpha band we observed stronger ERD for the target in the last two time windows; at electrodes CP5, CP6, P3, PZ, P4 (Monte Carlo *p* = 0.014) from 400 to 600 ms and at electrodes C3, Cz, CP5, CPz, CP6, P3, PZ, P4 (Monte Carlo *p* = 0.005) from 600 to 800 ms. Results indicated a differential processing in the alpha band between the two stimulus categories during this late time windows (cf. Fig 2, lower panel).

## Discussion

The aim of the present study was to investigate whether the use of angry prosody in the classical own name paradigm might be an alternative to presenting self-relevant salient information such as the voice of a family member. The utilization of a relative’s voice is specifically difficult to realize when working in clinical settings as intended for paradigms of the present kind. To this end, we looked at brain dynamics of well-established frequency bands (i.e. delta, theta and alpha) [27, 38] that have already been explored in an earlier version of this experimental paradigm [17]. We could observe that when passively listening to names, specifically, angry prosody induced specific changes in neural oscillatory activity. In particular, the angry voice led to stronger responses than the neutral voice but only in the delta range from 200 to 400 ms after stimulus onset. Previously, delta synchronization in response to emotional stimuli has been associated with the allocation of attention and detection of motivationally salient stimuli [30, 39]. This notion is well in line with our findings suggesting the presentation of angry prosody may have triggered automatic orienting of attention and thus preparation for further processing of potentially relevant stimuli. Surprisingly, in the passive condition, the effect was strongly restricted in time and we did not find significant effects of name or voice in other frequencies suggesting that the angry voice might trigger an initial orienting response but is not strong enough to evoke further changes in cognitive processing. It has to be mentioned that in the present study we did not specifically asked to pay attention to the emotional content of the presented stimuli; therefore the angry voice might have activated automatic attention orienting but without a clear discrimination of the emotional content. However, we previously used voice of relatives [17] and found differences in the alpha band with processing advantages for familiar and self-relevant stimuli. We suggested that these effects may have been related to long-term memory access and the reactivation of memory content. In the present study, it may have been the case that, despite being emotionally salient, angry voice stimuli took a different and more unconscious processing route. Also recently published data [31] revealed additional effects in the theta and alpha range (here only indicated as a trend, cf. Fig 1 and S1 Fig), which we do not see in the current analysis using a stricter statistical approach. Interestingly, Blume and colleagues (2016) were able to show that the subject’s own name as well as angry voice stimuli remained salient during light N2 sleep, which they specifically related to oscillatory responses in the delta and theta frequency range.

In the active condition, we were able to replicate our previous findings [17]. The current results indicate robust differences between target and non-target names in all frequency bands analysed, although with different temporal dynamics. In the delta range, the difference between target and non-target stimuli was evident from 200 to 800ms which is well in line with the interpretation of delta synchronisation indexing sustained attention while focusing on a specific salient target [40–43]. Likewise, the increase in theta band, in response to the occurrence of target stimuli was also evident from about 200 ms, however only persisting until 600 ms. Theta synchronization in this case is probably related to attentional processing and enhanced working memory engagement triggered by the detection of the target [27, 43–45]. As in the previous version of the paradigm [17], we also found a significant desynchronization in the alpha band in response to target presentation, which was only evident in the last two time windows, i.e. from 400 to 800ms. Previous studies that have found alpha desynchronization to vary as a function of task demands as well as the involvement of alpha oscillatory activity in general attentional processing [27]. Moreover, alpha synchronization has been shown to indicate inhibitory processes that are involved in successful stimulus processing [46]. From this perspective, our results may be explained by a release of inhibition after successful matching of the target name with memory contents as indicated by theta synchronization [28].

In conclusion, for the active paradigm we replicated previous findings and better characterized the temporal dynamics of target detection involving rapid delta and theta oscillatory EEG responses to the target stimulus as well as later alpha desynchronization reflecting release of inhibition following successful target detection. In the passive condition, the angry prosody interestingly only evoked an early delta response, which might reflect initial deployment of attentional resources to motivationally relevant stimuli. We think that the results render the paradigm interesting for the assessment of consciousness in non-communicative clinical groups and especially the high-end of the DOC spectrum. Whereas the passive part of the paradigm can only capture more automatic stimulus processing abilities the active part is tuned to identify non-communicative patients which can use their brain to indicate instruction following and consequently intact volition. Future studies are awaited that test whether this kind of paradigms can be applied in everyday clinical practice.

## Supporting Information

S1 Fig Topographic distribution of different frequency during the passive condition. Topographic maps depict the topographic distribution for delta, theta and alpha frequency band during the passive condition from 0 to 800 ms after stimulus onset. Subject’s Own Name (SON), Unfamiliar Name (UN) scalp distribution for the two conditions and p value distribution (right) are depicted. Note that no significant electrodes survived after cluster correction. Negative values indicate desynchronization (ERD) and positive indicate synchronization (ERS) respect to the baseline (from -700 to -200 ms before stimulus onset).
(TIF)

S2 Fig Topographic distribution of different frequency during the passive condition. Topographic maps depict the topographic distribution for delta, theta and alpha frequency band during the passive condition from 0 to 800 ms after stimulus onset. Angry Voice Subject’s Own Name (AV_SON), Neutral Voice Subject’s Own Name (NV_SON) scalp distribution for the two conditions and p value distribution (right) are depicted. Note that no significant electrodes survived after cluster correction. Negative values indicate desynchronization (ERD) and positive indicate synchronization (ERS) respect to the baseline (from -700 to -200 ms before stimulus onset).
(TIF)

S3 Fig Topographic distribution of different frequency during the passive condition. Topographic maps depict the topographic distribution for delta, theta and alpha frequency band during the passive condition from 0 to 800 ms after stimulus onset. Angry Voice Unfamiliar Name (AV_UN), Neutral Voice Unfamiliar Name (NV_UN) scalp distribution for the two conditions and p value distribution (right) are depicted. Note that no significant electrodes survived after cluster correction. Negative values indicate desynchronization (ERD) and positive indicate synchronization (ERS) respect to the baseline (from -700 to -200 ms before stimulus onset).
(TIF)

S4 Fig Example of auditory stimuli included in the study. (ZIP)
