# Supplementary figures and images for: The Voice of Anger: Oscillatory EEG Responses to Emotional Prosody
Source: PLoS One. 2016 Jul 21;11(7):e0159429. doi: 10.1371/journal.pone.0159429 (PMC4956258; doi:10.1371/journal.pone.0159429)

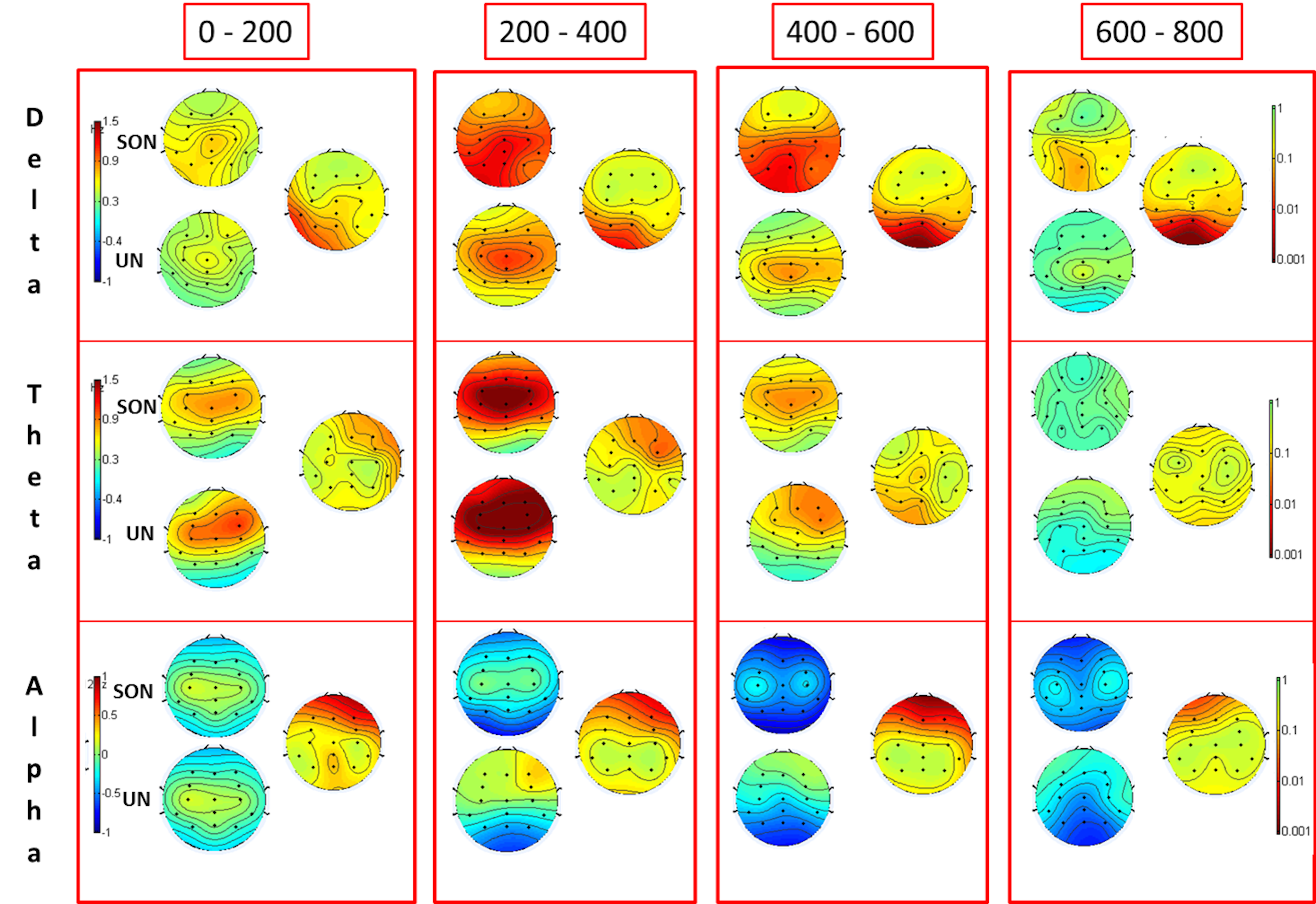

Supplement: S1 Fig — Topographic maps depict the topographic distribution for delta, theta and alpha frequency band during the passive condition from 0 to 800 ms after stimulus onset. Subject’s Own Name (SON), Unfamiliar Name (UN) scalp distribution for the two conditions and p value distribution (right) are depicted. Note that no significant electrodes survived after cluster correction. Negative values indicate desynchronization (ERD) and positive indicate synchronization (ERS) respect to the baseline (from -700 to -200 ms before stimulus onset). (TIF) [file pone.0159429.s001.tif]

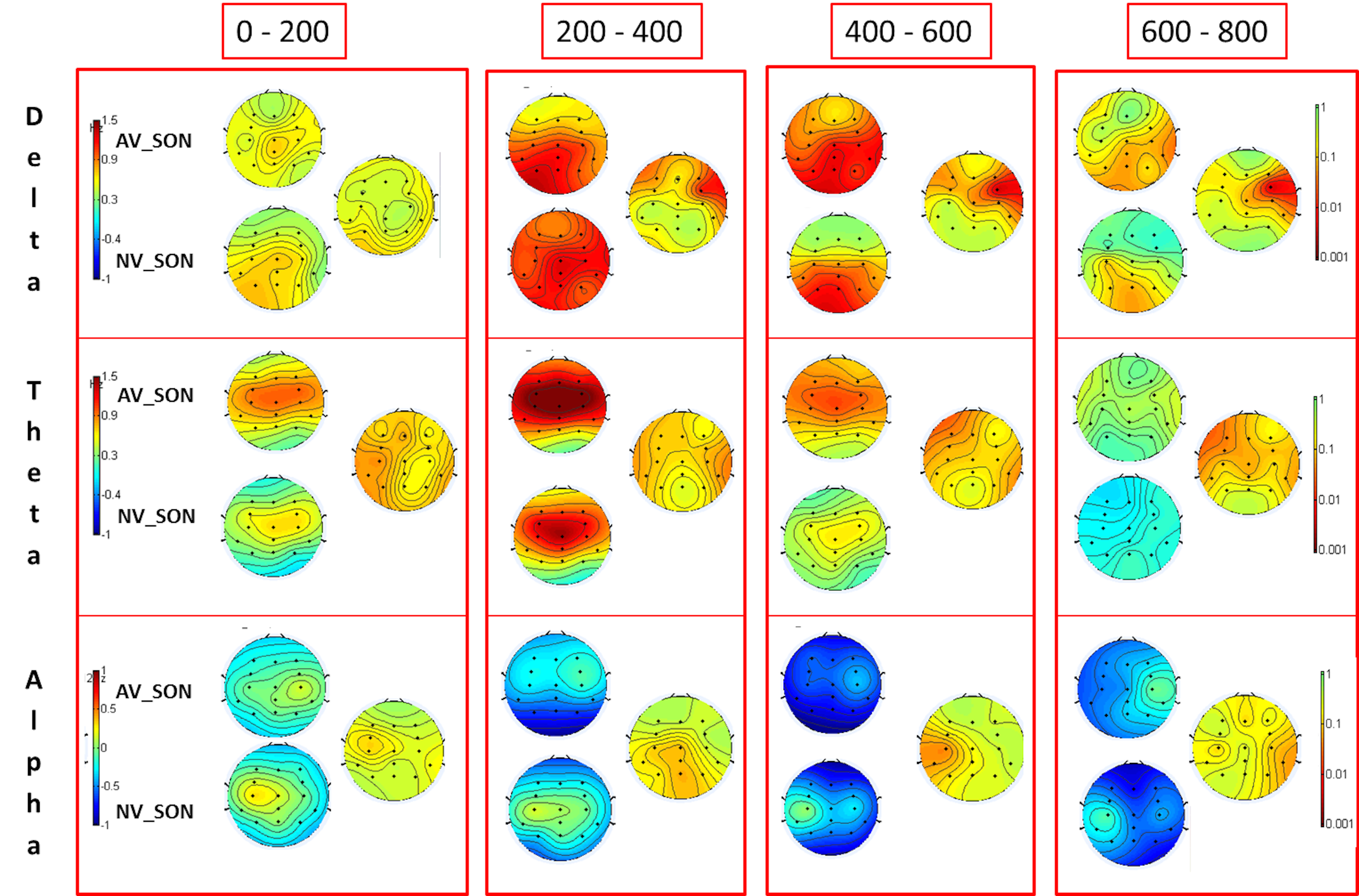

Supplement: S2 Fig — Topographic maps depict the topographic distribution for delta, theta and alpha frequency band during the passive condition from 0 to 800 ms after stimulus onset. Angry Voice Subject’s Own Name (AV_SON), Neutral Voice Subject’s Own Name (NV_SON) scalp distribution for the two conditions and p value distribution (right) are depicted. Note that no significant electrodes survived after cluster correction. Negative values indicate desynchronization (ERD) and positive indicate synchronization (ERS) respect to the baseline (from -700 to -200 ms before stimulus onset). (TIF) [file pone.0159429.s002.tif]

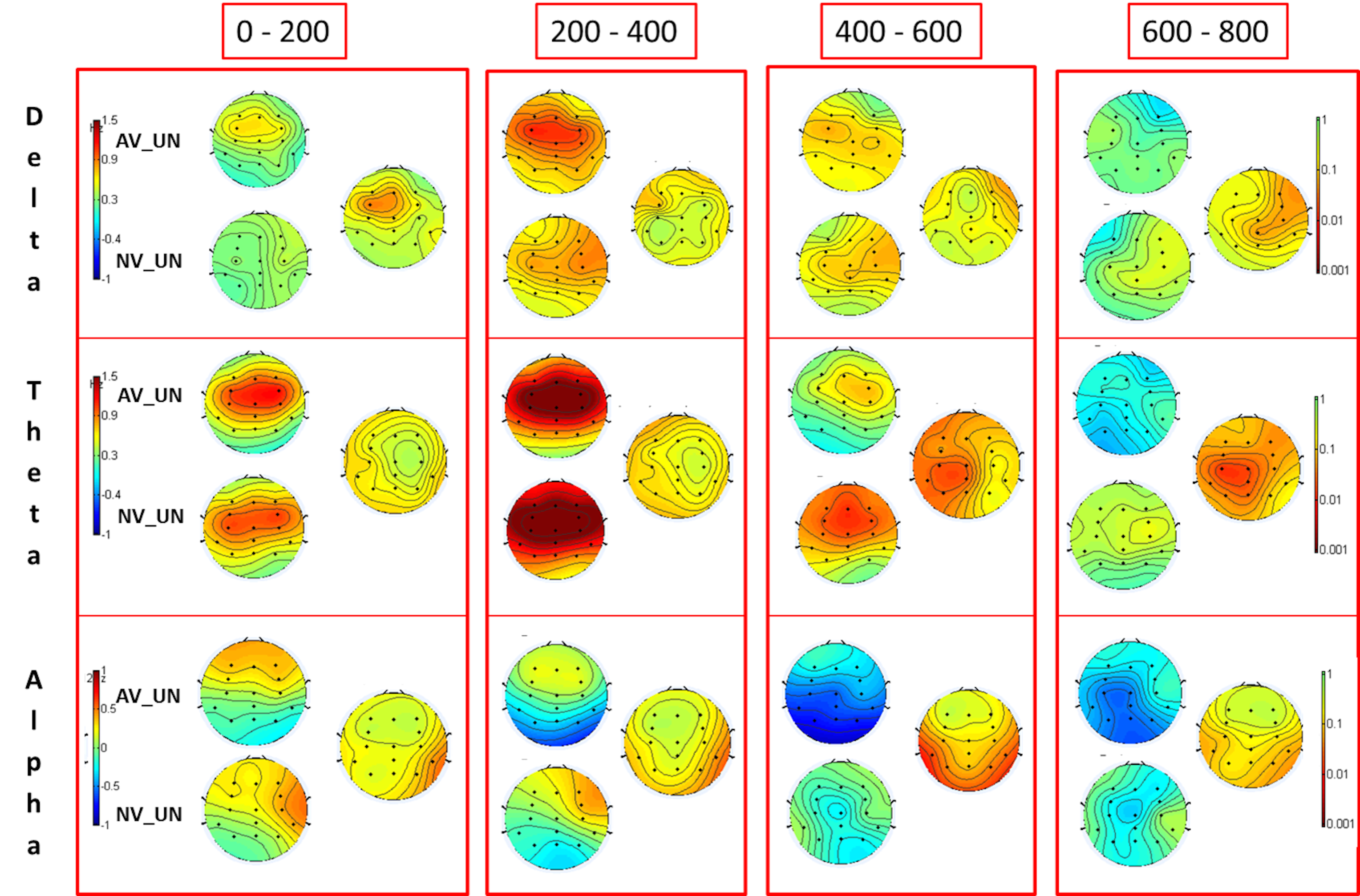

Supplement: S3 Fig — Topographic maps depict the topographic distribution for delta, theta and alpha frequency band during the passive condition from 0 to 800 ms after stimulus onset. Angry Voice Unfamiliar Name (AV_UN), Neutral Voice Unfamiliar Name (NV_UN) scalp distribution for the two conditions and p value distribution (right) are depicted. Note that no significant electrodes survived after cluster correction. Negative values indicate desynchronization (ERD) and positive indicate synchronization (ERS) respect to the baseline (from -700 to -200 ms before stimulus onset). (TIF) [file pone.0159429.s003.tif]
